# Neurobiology of anesthetic-surgical stress and induced behavioral changes in dogs and cats: A review

**DOI:** 10.14202/vetworld.2021.393-404

**Published:** 2021-02-11

**Authors:** I. Hernández-Avalos, E. Flores-Gasca, D. Mota-Rojas, A. Casas-Alvarado, A. E. Miranda-Cortés, A. Domínguez-Oliva

**Affiliations:** 1 Department of Biological Sciences, Clinical Pharmacology and Veterinary Anesthesia, Faculty of Higher Studies Cuautitlán, Universidad Nacional Autónoma de México, State of Mexico 54714, Mexico; 2 Department of Veterinary Surgery, Faculty of Higher Studies Cuautitlán, Universidad Nacional Autónoma de México, State of Mexico 54714, Mexico; 3 Neurophysiology of Pain, Behavior and Assessment of Welfare in Domestic Animals, DPAA, Universidad Autónoma Metropolitana, Mexico City 04960, Mexico; 4 Master in Agricultural Sciences. Animal Welfare, Universidad Autónoma Metropolitana, Mexico City 04960, Mexico

**Keywords:** cats, dogs, neurobiology, pain, stress, welfare

## Abstract

The anesthetic-surgical stress response consists of metabolic, neuroendocrine, hemodynamic, immunological, and behavioral adaptations through chemical mediators such as the adrenocorticotropic hormone, growth hormone, antidiuretic hormone, cortisol, aldosterone, angiotensin II, thyroid-stimulating hormone, thyroxine, triiodothyronine, follicle-stimulating hormone, luteinizing hormone, catecholamines, insulin, interleukin (IL)-1, IL-6, tumor necrosis factor-alpha, and prostaglandin E-2. Behavioral changes include adopting the so-called prayer posture, altered facial expressions, hyporexia or anorexia, drowsiness, sleep disorders, restriction of movement, licking or biting the injured area, and vocalizations. Overall, these changes are essential mechanisms to counteract harmful stimuli. However, if uncontrolled surgical stress persists, recovery time may be prolonged, along with increased susceptibility to infections in the post-operative period. This review discusses the neurobiology and most relevant organic responses to pain and anesthetic-surgical stress in dogs and cats. It highlights the role of stress biomarkers and their influence on autonomous and demeanor aspects and emphasizes the importance of understanding and correlating all factors to provide a more accurate assessment of pain and animal welfare in dogs and cats throughout the surgical process.

## Introduction

Stress is defined as an unconscious response to tissue damage. It may manifest as autonomic, metabolic, hormonal, immunological, and neuroendocrine changes provoked by injury or trauma [1,2]. When an organism’s central nervous system (CNS) perceives a potentially harmful stimulus, it responds through defense mechanisms that involve modifications of both behavior and the autonomic nervous system to restore homeostasis. This process is characterized by activation of the hypothalamic-pituitary-adrenal axis (HPA) and the sympathetic nervous system (SyNS), along with an increase in the concentrations of adrenocorticotropic hormone (ACTH) and cortisol [3,4].

The consequences of prolonged exposure to stressors include sensitization to pain, longer post-surgical recovery, and in some cases, sepsis, or delays in healing [5]. After surgical intervention, central stimulation is triggered through afferent nerve fibers that activate both the HP and sympathetic-adrenal-medullary axes [6], both of which transmit information to the paraventricular nucleus (NPV) of the hypothalamus through a network that includes areas of the amygdala, the nucleus of the bed of the stria terminalis, and the prefrontal cortex [7,8]. The NPV initiates the sequence of events by secreting corticotropin-releasing hormone (CRH) and vasopressin (VP) into the hypothalamic portal system, which induces ACTH production in the adenohypophysis with the subsequent stimulation of receptors in the fascicular area of the adrenal cortex to release glucocorticoids, mainly cortisol and corticosterone, into the bloodstream. Together, these events constitute the “fight-or-flight” response and the basis of the neurobiology of acute pain [9,10].

This review discusses the basic neurobiology and physio-metabolic outcomes in dogs and cats that suffer acute pain caused by surgical trauma. Highlighting the role of stress biomarkers and their influence on autonomic and behavioral aspects, emphasizing the importance of understanding and correlating all factors to provide a more accurate assessment of pain and animal welfare in dogs and cats throughout the surgical process.

## Concept of Surgical Stress

Surgical stress has been defined as “the biological response to factors that alter or threaten homeostasis.” The main stressors associated with surgical procedures are physical (e.g., tissue damage, impingement, and perception of pain) and chemical (application of antiseptics to the affected area) factors that promote specific reactions as a compensatory mechanism to prevent secondary damage and increase the availability of the substrates that essential organs require [6,11]. This process generates physiological changes that correlate with stressful states and/or disease [12].

Some authors consider that surgical lesions are accompanied by a series of specific reactions as a compensatory mechanism to prevent secondary damage and increase the availability of the substrates that essential organs require. This process generates physiological changes that correlate with stressful states [6].

Certain studies suggest that applying any anesthetic can potentially modify physiological responses to surgical procedures due to (i) hypnosis induced; (ii) alterations of the organic functions of the cardiovascular, respiratory, digestive, and neuroendocrine systems; (iii) metabolic changes; and (iv) affectations of immunological homeostasis since the endocrine, nervous, and immunological processes involved have physically and functionally related interactions [6,13-15].

## Neuroendocrine Response of the HPA

**Figure-1:**
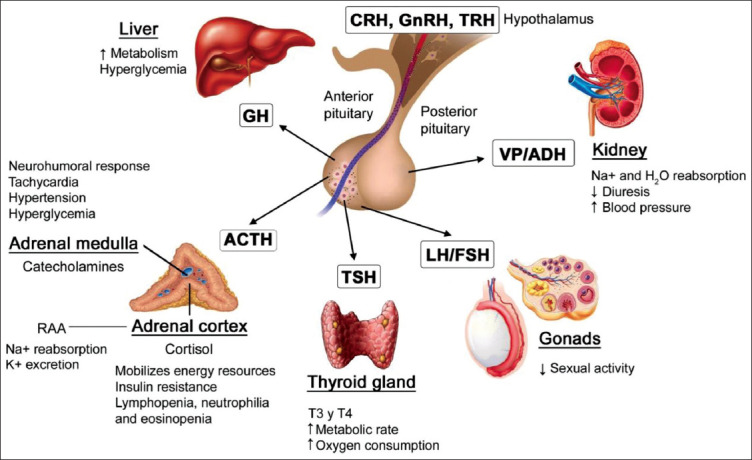
The hypothalamic-pituitary-adrenal axis and the response to surgical stress. ACTH=Adrenocorticotropic hormone, CRH=Corticotropin-releasing hormone, FSH=Follicle-stimulating hormone, GH=Growth hormone, GnRH=Gonadotropin-releasing hormone, K+=Potassium, LH=Luteinizing hormone, Na+=Sodium, RAA=Renin-angiotensin-aldosterone, TRH=Thyrotropin-releasing hormone, TSH=Thyroid-stimulating hormone, VP=Vasopressin [Source: 1,8,14,16].

One consequence of activation of the HPA is a greater secretion of catabolic hormones. This triggers a cascade of metabolic processes that degrade proteins, fats, and carbohydrates [1,12,16]. This cascade may contribute to the development of hypovolemia and painful states that can set off a systemic, post-trauma neuroendocrine response by stimulating the efferent neurons around the wound site [8,17]. This response is characterized by the pituitary glands increase in hormone secretion and activation of the SyNS. The ACTH, growth hormone (GH), and antidiuretic hormone (cortisol and aldosterone) concentration levels increase, while the effects of others, such as thyroid-stimulating hormone (TSH), follicle-stimulating hormone (FSH), and luteinizing hormone (LH), may increase or decrease during neuroendocrine control (Figure-1) [1,8,14,16].

These changes aim to maintain an adequate volume of fluids in the body by counteracting the effect of the renin secreted by the juxtaglomerular cells as a reflex reaction to efferent sympathetic stimulation [1]. Renin and angiotensin-converting enzyme act on their respective substrates to produce the active form called angiotensin II, which raises blood pressure through arteriolar vasoconstriction and by stimulating aldosterone secretion to restore circulatory volume during hypovolemic states [14].

Plasma VP exerts a similar action during hypotension by causing reabsorption of Na+ and water, which decreases urine production and increases systolic blood pressure and mean arterial pressure (MAP). This hemodynamic regulation correlates with the degree of pain after stretching of the ovarian pedicle and soft-tissue management [18,19]. Neurosecretion of VP that goes on for 3-5 days, depending on the severity of surgical damage, is associated with complications in the immediate post-operative period [20,21].

## Role of Thyroid Hormones in Surgical Stress

The most important thyroid hormones are thyroxine (T_4_) and triiodothyronine (T_3_). Their secretion is controlled by the TSH [1]. Together with catecholamines (epinephrine and norepinephrine), these hormones stimulate oxygen consumption in most metabolically active tissue, except for organs such as the brain, spleen, and anterior part of the pituitary gland. T_4_ and T_3_ help regulates the metabolic rate by increasing the absorption of carbohydrates from the intestine and stimulating the central (CNS) and peripheral nervous system [22]. However, these hormones are suppressed by both exogenous and endogenous corticosteroids during surgery [23].

## Relationship of Catecholamines to Surgical Stress

Stimulation of the SyNS by anesthetic drugs or acute perioperative pain produces secretion of the corticotropin-releasing factor (CRF), which stimulates the release of ACTH. The main role of ACTH is to activate the adrenal cortex so that it secretes cortisol, corticosterone, and aldosterone. This mechanism is involved in the excretion of catecholamines during surgery after stimulation of the adrenomedullary region, playing an important role in the neurohumoral modulation of stress and nociception [11,24].

Other studies have shown that the actions of these neurotransmitters are controlled by the activation or stimulation of α-adrenergic receptors (sub-divided into α_1A_, α_1B_, α_1D_, α_2B_, and α_2C_) or β-adrenergic (sub-divided into β_1_, β_2_, and β_3_) that are found in catecholaminergic neurons [25]. Both types are sensitive to epinephrine and norepinephrine, but the responses they induce differ with the tissue and number of receptors present [21]. The main metabolic effect is performed by β_2_ receptors as they increase glycemia by promoting glycogenolysis and gluconeogenesis of hepatic and muscular origin. During stress, no glucose-6-phosphatase is available, so muscular tissue produces lactate instead of glucose. However, if the harmful stimulus does not persist, the liver will re-convert the lactate into glucose [6].

Cardiovascular effects such as hypertension and tachycardia occur when catecholamines interact with the α_2_ receptors that modulate the release of norepinephrine in the post-synaptic nerve endings as a result of epinephrine secretion in the adrenal medulla. This process affects the adenylyl cyclase system to increase cardiac contractility [26]. Finally, these receptors have been identified as components of the pain modulating pathway because the α_2_ type can inhibit adrenergic impulses [25].

## Cortisol

Cortisol is produced by the adrenal cortex. Its serum concentration increases in the perioperative period due to the release and stimulation of ACTH. High concentrations of both hormones were found during the immediate post-operative period when intraoperative analgesia was insufficient [27]. In addition, its ratio has been shown to be a clinical marker to detect adrenal response due to stress in pathologies such as feline idiopathic cystitis [28].

The primary metabolic effect of cortisol is mobilizing energy. Cortisol inhibits the use of glucose by cells in a reaction influenced by an apparent resistance to insulin. This is related to the direct response of β cells in the pancreas, which promotes gluconeogenesis in the liver and the use of glucose by somatic cells [29].

In the liver, glucocorticoids facilitate the ability to concentrate amino acids from extra-hepatic tissue (skeletal muscle) for subsequent degradation. This occurs because cortisol significantly enhances hepatic glutamic-pyruvic transaminase activity and subsequently increases the transamination of alanine to pyruvate and then lactate [26]. The release of catecholamines also increases the amount of pyruvate available for its later transformation into glucose [30].

In combination with catecholamines and GH, cortisol contributes to lipid metabolism by promoting the conversion of triglycerides into glycerol and fatty acids. These metabolites act as an important energy source in the perioperative period and are utilized for gluconeogenesis and eliminating fatty acids during ketogenesis. Fatty acids can be oxidized in the liver and muscle to be converted into ketone bodies or re-esterified [16,31].

## Related Gonadotropic Hormones: FSH, LH, and Testosterone

Sex steroids are known mainly for their role in fertility and reproduction, but during stressful events, they are also mediated by the HPA when it recognizes a nociceptive stimulus [32,33]. The effects of gonadocorticoids vary, but, in general, testosterone, LH, and FSH concentrations decrease during the first 5 post-operative days [32,34,35].

## GH

Like glucocorticoids, this hormone contributes to hyperglycemia by stimulating glucose production, but it also has an anti-insulin effect that inhibits glucose uptake and its use by somatic cells. GH activity differs from that of other hormones, such as cortisol and catecholamines, which stimulate gluconeogenesis and glycogenolysis for the subsequent generation of glucose, which eventually serves as an energy source [8]. Moreover, it creates energy reserves for high energy demand organs, such as neurons, under hypoglycemia conditions [36,37].

## Other Alterations Associated with the Neuroendocrine Response

### Glucose, glucagon, and insulin

In general, catecholamines and cortisol in the bloodstream enhance glycogenolysis and gluconeogenesis to increase glucose concentrations during the trans-surgical interval. “Stress hyperglycemia” is sometimes confused with diabetes in felines [38]. However, when it is a mere consequence of stress, it is associated with behavioral changes such as aggression or reluctance to handling [39]. Hyperglycemia persists due to the action of catabolic hormones and lowers insulin activity; the latter is reported as a key hormone for anabolism [1]. There are reports that insulin is a key hormone for anabolism, as it is synthesized in pancreatic β-cells after eating or when blood glucose and amino acid levels rise. GH promotes glucose uptake from striated skeletal muscle and adipose tissue through the conversion of glucose and triglycerides. In addition, it stimulates the formation of glycogen from glucose in the liver [40].

Both the induction of anesthesia and surgical stimulation cause insulin resistance due to the activation of α-adrenergic receptors and the inhibition of pancreatic α-cells to equalize catabolism in response to hyperglycemia [36,41].

Glucagon is another product secreted by pancreatic β cells that may occur at higher levels after major surgical interventions. This mediator stimulates hepatic glycogenolysis, increases gluconeogenesis from amino acids in the liver, and controls lipolytic activity. There is still wide debate on its effects because observations have shown that it does not contribute significantly to the hyperglycemic response [42].

### Lactate

After gluconeogenesis or glycogenolysis, a molecule of glucose through anaerobic glycolysis is transformed into two molecules of pyruvate to generate two molecules of adenosine triphosphate (ATP) that produces energy. Pyruvate can follow two metabolic pathways: In the presence of oxygen, it enters the Krebs cycle and creates 36 ATP molecules. In contrast, when the availability of blood oxygen is insufficient, or when the capacity of the pyruvate dehydrogenase enzyme is exceeded, it results in the creation of lactate, a biofuel present in the skeletal muscles, brain, heart, kidney, and liver [29,43,44].

Thus, lactate and hyperlactatemia can be considered reliable biomarkers of hypoxia, hypoperfusion, and anaerobic glycolysis [45], and its presence is indicative of an accelerated state of glycolysis posterior to SyNS activation to ensure a sufficient supply of bioenergy [46]. However, hyperlactatemia has also been reported with strenuous exercise [47] or mountain climbing where oxygen input is low. Hyperlactatemia is not a necessary condition, even though pO_2_ values of 25 mmHg have been reported. A similar situation has been described in patients with sepsis [48]. Garcia-Alvarez *et al*. [45] suggested that hyperlactatemia should not be seen as a biomarker of hypoxia or anaerobic glycolysis but rather as a major component of the stress response during activation of the HPA.

## Cardiovascular Changes associated with the Neuroendocrine Response

Recent work has shown that hypertension and tachycardia reflect SyNS activity after nociceptive stimulus caused by trauma or stress [1,14,30]. This was evaluated during elective ovariohysterectomy in bitches, where increased heart rate and blood pressure were observed when the harmful stimulus was most intense [20].

Thrasher *et al*. [49] described high concentrations of plasma VP after a period of hypotension in the presence of harmful stimuli. This hormone is a neuropeptide that can be altered by high adrenal corticosteroid levels, so its presence determines the duration of the effects of corticosteroids. VP acts on kidneys and the juxtaglomerular cells, promoting water retention and concentrated urine associated with surgical trauma, hypovolemia, and activation of pressure and volume receptors in the CNS [49,50].

Another mechanism in the kidney is the stimulation of juxtaglomerular cells. These cells synthesize, store, and secrete renin, the enzyme that catalyzes angiotensin II production and, subsequently, the release of aldosterone into the adrenal cortex, which is responsible for promoting the reabsorption of Na+ and water in the distal tubules, which means that it participates in regulating the blood [51].

During surgical procedures, blood oxygen pressure (SaO_2_ and SpO_2_) decreases. This is most evident when patients do not receive pre-oxygenation, which triggers a decrease in oxidative metabolism with the activation of the anaerobic glycolysis pathway. Here, lactic acid accumulation and the development of lactic acidemia increase progressively, causing intracellular damage and cell death. In the post-operative period, the effects of hypoperfusion and hypoxia as causes of reduced blood flow become more evident, while high lactate levels, metabolic acidosis, and increased free fatty acids are also present [16].

## Immune Response

After a harmful event, such as a surgical procedure, the immune system may be either activated or depressed according to the type of stress-induced. This response is mediated by immunological mediators such as cytokines or interleukins (ILs), low molecular weight proteins produced by the activity of leukocytes, fibroblasts, and endothelial cells [52]. Cytokines cause pro-inflammatory responses. They include IL-1, IL-6, and tumor necrosis factor-alpha (TNF-α), which have an effect on metabolism and play a key role in regulating cardiovascular, endocrine, and neuronal activity [53]. IL-1 is a cytokine synthesized by monocytes and leukocytes into two isoforms (IL-α and IL-β, predominantly the β isoform) that perform a regulatory function of myelopoiesis [54]. It is considered an endogenous pyrogen that raises body temperature and increases prostaglandin E-2 synthesis, whose action on the hypothalamus causes fever and anorexia [55].

TNF-α, in turn, is produced by monocytes, macrophages, Kupffer cells, mast cells, and lymphocytes [56] and is related to metabolic effects. This is due to increased transmembrane glucose transport and glycolysis function that, together with IL-1, promote the proteolysis of skeletal muscle and the release of amino acids by producing serum iron and zinc deficiency through the release of the protein to which these minerals tend to bind. Thus, these cytokines contribute to states of deep anorexia and the development of cachexia [52]. In addition, prolonged exposure to excessive concentrations of these two cytokines has a negative inotropic effect on the myocardium that can cause left ventricular dysfunction, increase nitric oxide levels, and promote vasodilation [56].

**Figure-2:**
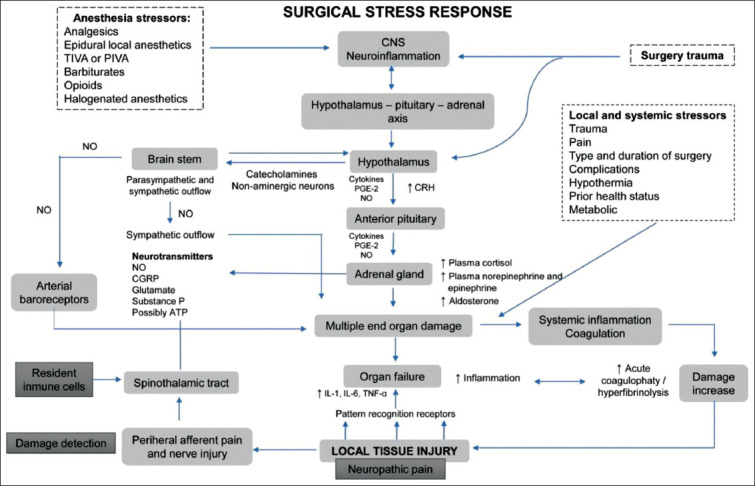
Schematic of the neurobiological interaction between the hypothalamic-pituitary-adrenal axis, the metabolic changes, and the immune system after anesthetic-surgical stimuli. CNS=Central nervous system, CGRP=Calcitonin gen-related peptide, CRH=Corticotrophin-releasing hormone, IL=Interleukin, NO=Nitric oxide, PIVA=Partial intravenous anesthesia, PGE=Prostaglandin, TIVA=Total intravenous anesthesia, TNF=Tumor necrosis factor. Surgical stress, caused by tissue injury or anesthetic drugs, activates the HPA and hence the release of catecholamines and cortisol (known as stress hormones) in the adrenal gland. This action is controlled by factors such as CRH, VP, and ACTH in the pituitary gland. Under pathological states or persistent pain, the enhanced activity of cytokines (IL, TNF-α), PG, and NO can contribute to ischemia, inflammation, and coagulopathies. Together with the neurotransmitters coming from the CNS and spinal cord, this may result in additional damage [Source: 1,3,5,53,56,58].

IL-6, which is biosynthesized by monocytes and macrophages, has been detected in cerebrospinal and synovial fluids during bacterial and viral infections of the CNS and inflammatory arthritis [52,57]. In contrast to IL-1 and TNF-α, the participation of IL-6 depends on the magnitude of the tissue injury and control of the immune response. Its plasma concentration increases rapidly in patients undergoing surgery, but the secretion rate can be influenced by anesthetic drugs and endogenous corticosteroid levels (Figure-2). Experimental evidence suggests that controlling the immune system and its mediators may mitigate the consequences of traumatic injuries and, hence, decrease perioperative morbidity and mortality [51]. Table-1 summarizes the main immunomodulatory effects of some sedatives[58-79], local analgesics, and anesthetics commonly used in dogs and cats.

**Table-1:** Immunomodulatory effects of the most commonly used sedatives, local analgesics, and anaesthetics for dogs and cats.

Drug	Immunomodulatory effects	References
Acepromazine	Antioxidant effect decreases ROS.	[58]
	Oxidation-reduction reactions are reduced in bacteria like *Mycobacterium* spp.	[59]
Dexmedetomidine	Modulation of TLR_4_ and NFjB, pro-inflammatory cytokines decrease. Phagocytosis is stimulated. Reduces TNF_α_, TLR, and IL-6.	[60] [61] [62]
Ketamine	NK cells, neutrophil chemotaxis, macrophages, and cytokines are suppressed. Thromboxane B2, TNF_α_, IL-1β, and IL-6 are reduced. Stabilizes the release of TLR_4_ and NFjB.	[63] [64] [65] [66]
Lidocaine	Reduces phagocytes, neutrophil adhesion, ROS, and PG production. Stabilization of endothelial membrane.	[67] [68] [69]
Midazolam	Has an anti-inflammatory and immunosuppressive effect. Decreases COX_2_ and iNOS, phagocytes. Lymphocyte proliferates and there is an alteration in neutrophil apoptosis.	[70] [71] [72]
Morphine	Interferes with antigen presentation; increases lymphocyte apoptosis and alters B lymphocytes differentiation. Reduced phagocytosis and NK activity. Activates T lymphocytes.	[73] [74] [75]
Propofol	The antioxidant effects of PGE2 decrease. Inhibits neutrophil and macrophage phagocytosis, as well as ROS production. NK function is stimulated.	[76] [77]
Thiopental	Suppression of T lymphocyte function. A reduction of platelet tissue factor and TNF production. Inhibits the expression of lipopolysaccharide-induced tissue factor.	[78] [79]

COX=Cyclooxygenase, iNOS=Inducible nitric oxide synthase, NF=Nuclear factor, IL=Interleukin, NK=Natural killer, PG=Prostaglandin, ROS=Reactive oxygen species, TLR=Toll-like receptor, TNF=Tumour necrosis factor

## Involvement of Glucocorticoids in the Immune Response

Glucocorticoids produce a “stress leukogram” characterized by eosinopenia due to intravascular lysis, sequestration in the liver and spleen, decreased release from bone marrow, and migration to the lymphoid tissues that generate glucocorticoids. Lymphopenia is also present due to the sequestration in tissues and the adherence of circulating lymphocytes to the endothelial cells. Glucocorticoids also stimulate the migration of neutrophils from the bone marrow to the bloodstream and attenuate their transit to other compartments, generating neutrophilia with increasing concentrations of mature and immature neutrophils [80]. In cats, the variations in leukocytes are usually greater than in dogs (up to 4 times the reference values), leading to physiologic leukocytosis [81].

In general, pain and surgical stress suppress the immune system and predispose animals to infection, and extended hospital stays because immunosuppression promotes inflammation and delays healing [82]. Figure-2 shows a schematic summary of the neurobiology of stress related to anesthetic-surgical stimuli.

While all these biological events maintain a close relationship to possible behavioral changes that patients may show during clinical evaluations, no single physiological or behavioral parameter can serve as a specific indicator to raise suspicions that an individual animal is suffering from surgical stress. Although most or all of the responses mentioned above may be present, it is essential to correlate all the neurobiological assessments performed during both the intra- and post-operative periods [82].

## Behavioral Changes Associated with a Predominance of Sympathetic Tone that Indicates Hemodynamic Reactivity due to Pain and Surgical Stress

Pain is the main factor that triggers changes in the neurobiology of stress after surgery since it is responsible for the neuronal and biochemical feedback through mediators such as substance P, bradykinin, histamine, glutamate, potassium ions, and the calcitonin gene-related peptide [11]. These substances, known together as the “inflammatory soup,” have been associated with pain or stress in animals, where studies have recognized alterations that can affect the state of alertness and performance of learning and memory [83].

Research has determined that intensive sensory stimulation (i.e., somatic, visual, and acoustic inputs in the brain) activate the locus coeruleus, limbic regions (hypothalamus, hippocampus, and amygdala), and cerebral cortex, causing adaptive responses to acute stress with enhanced neuronal activity due to the excitatory properties of ACTH and CRF that alter periods of vigilance and agitation and results in reactions such as startling, anxiety, fear, and, in some animals, euphoria [11]. Some researchers interpret these behavioral changes in dogs and cats as indicators of stress [10].

The hemodynamic reactivity associated with pain is generated by sympathetic-adrenergic and motor activation. Once the painful impulse reaches the spinal cord, the dorsal horn neurons establish synapses with somatomotor neurons and sympathetic preganglionic neurons. Stimulation of these neurons causes changes in the neurological, cardiovascular, respiratory, digestive, urinary, muscular, and endocrine systems [84]. Behavioral alterations associated with painful processes include temperament, vocalizations, posture, and locomotion, and its manifestation depends on factors such as the species, breed, health, and age [85,86].

Stimulation of the cardiac, respiratory, and endocrine centers occurs once the nociceptive information reaches the brainstem, thalamus, and hypothalamus. Alterations in the cardiovascular system result from sympathetic activity and release of catecholamines that cause hypertension due to peripheral vasoconstriction, tachycardia, increased myocardial contractility, and systemic vascular resistance. This, in turn, generates a high demand for oxygen and elevated oxygen consumption by the myocardium [84]. An increase of 20% in the sympathetic tone from the baseline heart rate, respiratory rate, and MAP is usually associated with mild-to-moderate pain. In comparison, a 50% increase is interpreted as severe pain that requires rescue analgesic therapy [86,87]. In addition, the so-called Virchow triad fosters the onset of thromboembolic phenomena. Likewise, the persistence of peripheral vasoconstriction can generate acidosis due to the low oxygen supply to tissues [82,87].

Changes in respiratory patterns appear after stimulation of the respiratory centers. Diaphragmatic dysfunction and reflex contractures of the thoracoabdominal musculature limit the expansion of the thoracic cavity, raising the risk of respiratory infections and retention of secretions. Hyperventilation initially occurs, but this soon turns into hypoventilation associated with hypoxemia, hypercapnia, tachypnea, and wheezing. In addition, to the increased oxygen demand induced by catecholamines, hypoventilation may worsen respiratory acidosis [84,88].

When nociceptive information reaches the somatosensory cortex, it produces the so-called cortical responses, manifested as behavioral and psychological actions that determine the animal’s reaction. This wide variety of signs is usually cited in pain scale evaluations for the clinical recognition of pain or states of surgical stress [84]. In general, the position of the body, facial changes in the eyes, pupils, and orientation of the ears and whiskers, demeanor, vocalizations (such as cries, whimpers, and growls), appetite (hyporexia or anorexia), urination, grooming, and social behavior are considered to assess the level of discomfort and can differ between species [85]. There is a prevalence of antalgic postures in dogs such as “prayer posture”, aggression, restriction of movement, star-gazing behavior, excessive licking, crouching, hypervigilance, intermittent vocalization, shivering, and submissive attitude [89]. However, cats tend to be depressed with a hunched posture, muzzle tensed, squinted eyes, ears flattened outwards, whiskers straight and forward, and head below the shoulder line. They also show sudden freezing, nibbling the injured area, tail flicking, hiding at the back of the cage, vocalization when approached (groan, hiss), and guarding behavior with prolonged recumbency [90,91]. Decreased pain tolerance can also cause a delay in the growth rate of young animals [86-88,92]. Some of the common stress indicators are listed in Table-2[84,85,88,89,91].

**Table-2:** Summary of the main physiological, behavioral, and laboratory parameters changes as a result of acute pain and surgical stress [84,85,88,89,91].

Physiological changes	Behavioral changes	Changes in biochemical parameters
Tachycardia Hypertension Cardiac arrhythmias Tachypnea Superficial respiratory pattern Pale mucous membranes Mydriasis Sialorrhea Hyperglycemia	Vocalization Look and lick the affected area Alteration of the facial expression Self-mutilation Muscle stiffness or weakness Restlessness and anxiety Apathy and inactivity Aggression, fear, and depression Stereotypes Anorexia or hyporexia Reduction of grooming Prayer posture Sleep disorders	↓ PaO_2_ ↓ PaCO_2_ ↓ HCO_3_ ↑ H+ ↑ Cortisol ↑ Lactate ↑ Glucose

## Biomarkers and the Development of Tools to Assess Pain and Surgical Stress

Several indicators have been described in the literature to determine stress in animals under various circumstances, usually by measuring pain [93,94]. The biomarkers most often used are cortisol and glucose levels, two substances involved in the adrenocortical response. However, some studies classify these as non-specific because their secretion and synthesis can be affected by administering drugs (e.g., fentanyl) or other factors, such as anxiety [15,93,94].

In an attempt to design a simple clinical parameter that could correlate physiological reactions with surgical stress, developed a tool that makes it possible to evaluate the variability of heart rate and signs of absolute amplitude from plethysmography, which indicates the degree of tissue perfusion and its relationship to catecholamine, physiological reactions that occur during surgical stress [95]. However, this method has not yet shown high sensitivity or specificity for detecting stress or pain [96]. The pleth variability index (PVI) is a method used to monitor fluid responsiveness and hemodynamic optimization and is associated with tissue perfusion and perioperative serum lactate levels. In dogs undergoing surgery, PVI-guided fluid therapy significantly lowered lactate levels [97]. However, between species, its levels are not always consistent with the severity of the process, and cats with a slight increment show higher mortality due to the exponential increase with increased compromise rather than the linear fashion observed in dogs [46].

Another study evaluated blood pressure, plasma VP concentrations, and urinary noradrenaline/creatinine levels, but it concluded that, despite evidence of elevated indices for these three parameters, there was no difference in, or relationship to, harmful stimuli, such as the removal of ovaries [20].

Srithunyarat *et al*. [12] evaluated the association among chromogranin A, catestatin, and vasodilatation in dogs undergoing elective surgery (these are biomarkers associated with stress in humans that have shown high sensitivity). Their results documented significant differences in these three biomarkers’ plasma levels after surgery and concluded that these markers could be used effectively to evaluate stress and painful stimuli in dogs subjected to harmful procedures. However, further studies are needed to corroborate their findings.

As described above, evaluations of nociception in anesthetized animals are based not only on detecting hemodynamic reactivity, defined as tachycardia and increased blood pressure but also on changes in respiratory patterns or locomotion. However, such modifications may not be specific to nociception and can be affected by anesthetic agents, clinical condition, and surgery [98]. For this reason, several methods have been proposed to more reliably quantify the nociception/anti-nociception balance by analyzing reflex pathways, pulsed photoplethysmography signals, pupillometry, and heart rate variability (HRV) [99-101].

**Figure-3:**
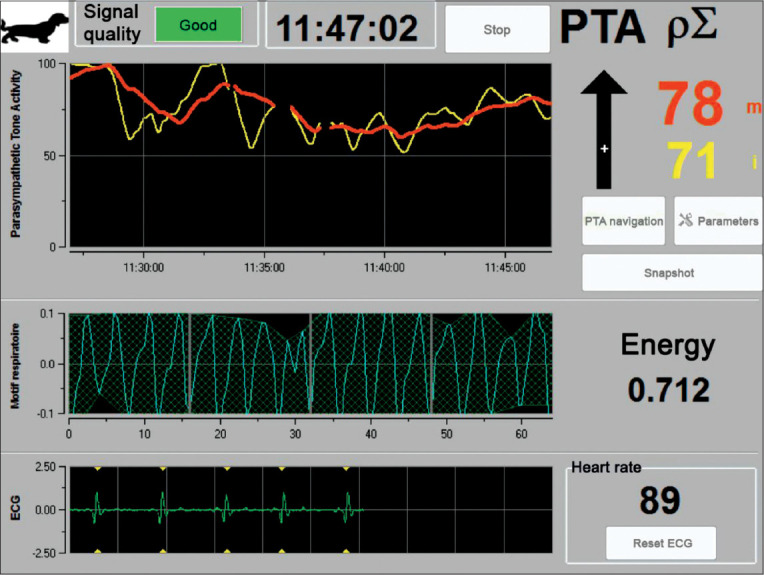
Parasympathetic tone activity index monitoring for the assessment of pain. PTA uses the electrocardiogram signal to evaluate heart rate variability as a non-invasive method for assessing the autonomic nervous system. High-frequency waves (0.15-0.5 Hz) are observed in this figure, which are related only to parasympathetic activity. In this case, it was concluded that the patient did not experience pain.

In human anesthesiology, the analgesia/nociception index, derived from HRV, is an index that reflects a relative parasympathetic tone and is considered a validated tool for detecting intraoperative nociception [102-105]. This technology has been adapted in veterinary medicine as the parasympathetic tone activity (PTA) index to study acute pain in dogs, cats, and horses. Both indices analyze HRV and reflect the sympathovagal balance [106]. Although they can be altered by premedication during surgery, they can predict hemodynamic reactivity [107] and indicate the analgesia/nociception balance [108,109]. A high value would indicate a high parasympathetic tone and the absence of nociception, and a low value would reflect a low parasympathetic tone and potential nociception (Figure-3) [98]. In conscious patients, PTA provided objective scores of the level of stress in cats during physical examination [110].

Furthermore, infrared thermography is another tool that evaluates perioperative stress and analgesia by measuring peripheral tissue microcirculation, which is altered during an acute injury [111]. Spectral entropy in tail clamping and the nociceptive withdrawal reflex in responsive cats are monitors designed to assess intraoperative nociception. However, few studies evaluate the level of surgical influence in animals [112].

As the evidence presented herein shows, we do not yet have a specific biomarker that effectively quantifies the surgical stress or pain that animals perceive; rather, it is necessary to integrate several biomarkers and evaluations to make an overall assessment. The current chemical markers and pain scoring systems have limitations regarding the species, the physiology, the anesthetic protocol, and the observational assessment. Therefore, parameters such as PTA index and infrared thermography could be considered as additional tools for a multimodal and more objective insight into surgical stress and pain for further studies.

## Conclusion

Stress is an adaptive response necessary for a favorable reaction to harmful stimuli through metabolic, endocrine, hemodynamic, behavioral, and immunological mechanisms. However, it is also well-known that sustained, constant, and uncontrolled states cause unwanted effects during the post-operative recovery phase. Both the veterinary surgeon and anesthesiologist involved must be intimately familiar with all aspects of this complex process to control or, if possible, prevent stress. It is also imperative to acknowledge the absence of a single specific biomarker capable of objectively evaluating the degree of pain or stress in animals. Studies on surgical-anesthetic stress should be carried out using a comprehensive analysis of physiological signs (heart rate, respiratory rate, blood pressure, and temperature) and biochemical markers (cortisol, catecholamines, lactate, glucose, and IL), in addition to the assessment of autonomous responses to noxious stimuli with newly developed tools such as the PTA index or HRV, and behavior-based pain scoring scales. The integration of these parameters could help veterinarians obtain a more accurate evaluation to prevent and manage the neurobiological and behavioral aspects of the perioperative period.

## Authors’ Contributions

IHA conceptualized, drafted, and supervised the final version. IHA, EF, and DM contributed to the original draft, data curation, investigation, writing, review, and editing of the manuscript. AEM worked on the methodology, writing, and editing of the review. AC and AD collected relevant literature, wrote, reviewed, and edited the manuscript. All authors have read and approved the final manuscript.
